# Aggressive squamous cell carcinoma of the cranium of a dog

**DOI:** 10.1186/s12917-021-02843-8

**Published:** 2021-04-06

**Authors:** Anna Łojszczyk, Wojciech Łopuszyński, Mateusz Szadkowski, Maciej Orzelski, Piotr Twardowski

**Affiliations:** 1 Department and Clinic of Animal Surgery, Laboratory of Radiology and Ultrasonography, University of Life Sciences, Głęboka 30, 20-612 Lublin, Poland; 2 Department of Pathological Anatomy, Faculty of Veterinary Medicine, University of Life Sciences, Głęboka 30, 20-612 Lublin, Poland

**Keywords:** Computed tomography, Squamous cell carcinoma, Diagnostic imaging, Aggressive osteolysis, Skull

## Abstract

**Background:**

The authors report a case of keratinized squamous cell carcinoma (SCC) in a 14-year-old dog with extensive cranial bone invasion. To our knowledge, this is the first description of such a case of cranial keratinized SCC with aggressive generalized osteolysis described in a dog.

**Case presentation:**

The 14-year-old dog was referred for radiological examination with suspicion of head trauma with clinical signs of head deformation, exophthalmos and nasal discharge. The skull radiographs showed a large osteolytic defect of the frontal bone and parietal bone in the region of the external sagittal crest. Findings from the skull CT scan included generalized osteolysis in the region of parietal bone, frontal bones, maxilla on the right side and the nasal bone including the dorsal nasal concha. In the area of bone loss, new soft tissue formation with multifocal foci of mineralization was visible. The ultrasound examination revealed hypoechogenic changes with hyperechoic foci consistent with mineralization and poor vascularization. The brain and ocular structures were without visible changes. Fine needle aspiration cytology (FNAC) was performed, and squamous cell carcinoma was suspected. After 3 months, the re-presented to the clinic. The dog became progressively listless, his appetite was decreased, and he became acutely blind. Follow-up skull CT scan revealed significant osteolysis, which affected a significant aspect of the cranium. All bone defects had been replaced by new 3.5 cm-thick soft tissue formations with multifocal small 1–2 mm areas of mineralization. There was no evidence of metastasis. Histological examination confirmed the suspicion of squamous cell carcinoma.

**Conclusions:**

This paper is the first report of cranial SCC in a dog causing extensive bone osteolysis. The lesions in this dog originated from the frontal and parietal bones including frontal sinuses.

There are variants of tumors that arise from squamous epithelium or resemble SCC in the skull. These examples include adenosquamous carcinoma and proliferating trichilemmal tumours. In addition, there is possible malignant transformation caused by papilloma viruses. In the veterinary literature, there is only one similar description of adenosquamous carcinoma in a cat with similar clinical manifestations. It is justified to suspect a process of neoplastic epithelial origin in all cases of aggressive and extensive skull bone lysis. This issue should be subject to further investigation.

## Background

Squamous cell carcinoma (SCC) is a malignant neoplasia originating from squamous epithelium [1, 2]. In dogs, it accounts for approximately 5% of all skin tumors, while in people, it accounts for 20% of skin malignancies [1, 3]. In the cranium, it is the most frequent epithelial tumor of the zygomatic arch and calvarium, especially in cats [4, 5]. SCC involves mainly non-pigmented, relatively hairless skin and nasal planum, although it may also appear at other sites covered with squamous epithelium, such as gingival, tonsillar and lingual sites [1, 6, 7]. It can also originate from the mucosal lining of the frontal sinus and nasal passage [1, 6–8]. In dogs, other locations of SCC, such as the skin of abdomen, limbs, perineum and digits, have also been reported [1]. In people, the most commonly affected site is the head and neck region [1, 7].

Ultraviolet light as well as ionizing radiation, exposure to chemical carcinogens, long-standing dermatoses, scars and other chronic lesions are important predisposing factors [2, 9–11]. Recently, the papilloma viruses have been mentioned as potential factors involved in SCC etiology as well [12–16]. Despite the fact that the patomechanism of viral infections and neoplastic transformations have not been clearly elucidated, there are speculations that immunosupresion may be one of the risk factors for SCC development [17]. Tumors of viral etiology could be invasive, and there is a high risk of metastases, which are regional (65%) and distant, especially in the lung (20%) [13, 14]. In these cases, marked infiltration and destruction of surrounding tissues is typical [18].

The disease manifestation in animals is similar to that in humans and differs depending on the SCC malignancy grade and location. SCC is mainly locally aggressive and shows rapid growth, but lymph node and distant metastasis are rare [1–3]. An exception is SCC located in the palatine tonsils, for which micrometastases are often found in regional lymph nodes as well as in the lungs when the primary tumor is diagnosed [15]. In contrast to most common primary bone tumors of the calvarium, including osteosarcomas and chondrosarcomas, bone is not often involved and it does not have the tendency to be extensive [1, 19].

The authors report a case of keratinized squamous cell carcinoma in a 14-year-old dog with extensive cranial bone invasion. To our knowledge, this is the first description of such a case of cranial keratinized SCC described in a dog.

## Case presentation

A 14-year-old male intact mixed-breed dog, was referred for radiological examination after visiting the Department and Clinic of Small Animal Surgery. The owners stated that the dog had escaped from the property in the evening and was absent for approximately 2 h. They suspected that the dog had suffered a head trauma event because he returned with a visible bruise on his forehead and was apathetic for a few days. After 4 weeks, the owners noticed slight skull deformation with coexistent exophthalmos. The dog presented to the clinic approximately 7 weeks after the trauma with evidence of head deformation, exophthalmos, and mild nasal mucopurulent discharge. The patient was in good physical condition and retained his appetite and friendly behavior. The dog showed no overt discomfort on palpation of the lesion. Temperature, heart rate, and respiratory rate were normal (respectively 37.8 C, 89 bmp, 27 breaths per minute).

**Fig. 1:**
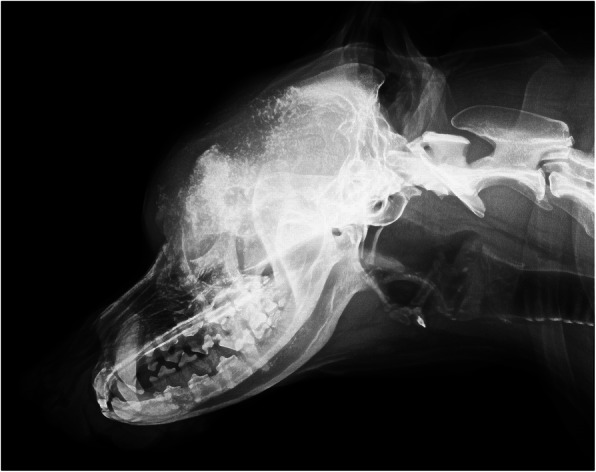
Radiographic examination of the skull with visible large osteolytic defect of the frontal and parietal bone in the region of the external sagittal crest

Skull radiographs were performed using an Arcoma direct radiography system (Arcoma 0130-FP, Aroma AB, Växjö, Sweden). Lateral and dorsoventral radiographs of the skull were obtained. The skull radiographs revealed a large osteolytic defect of the frontal bone (42 × 23 mm) and parietal bone in the region of the external sagittal crest (Fig. 1). In the region of the frontal bone, many foci of osteolysis and osteosclerosis with advanced bone remodeling were visible. The changes were mostly visible on the left side. Next, for the better evaluation of the bone changes, computed tomographic examination was proposed.

The dog was anaesthetized with Propofol (Scanofol 10 mg/ml, ScanVet, Gniezno, Poland), and a tomographic examination was performed with a Philips MX-16 slice unit (Philips Healthcare, Suzhou, PRC). The images were acquired with soft and sharp algorithms in soft tissue and bone windows. The soft tissue algorithm included unenhanced and contrast-enhanced phases with Iohexol iv injection at a dosage of 2 ml/kg BW (Omnipaque 300 mg/mL, GE Healthcare AS, Oslo, Norway). The image acquisition parameters were 120 kV, 200 mAs/slice, pitch 1.0069, slice thickness 1.0, slice increment 0.5 mm, collimation 16*0.75 and rotation time 0.75 s. Images were sent to the Philips IntelliSpace Portal (Philips Medical Systems Nederland B.V., Bests, The Netherlands) and to other computer units, where they were analyzed with the Horos program (GNU Lesser General Public License, Version 3, LGPL-3.0; Annapolis USA).

Findings from the skull CT scan included generalized osteolysis in the region of the maxilla on the right side and within the nasal bone, including the dorsal nasal concha (images not show). Almost a third of the dorsal aspect of the nasal cavity was occupied. The nasal septum was not visible in the dorsal part. The nasal bone on the left side in the caudal part was rebuilt and distorted with a large area of osteosclerosis. There were many areas of osteolysis within the dorsal aspect of the parietal bone and the area of the external sagittal crest, which were observed during radiography. In the area of bone loss, new soft tissue formation with multifocal foci of mineralization was visible. In the area of the parietal bone, the formations were 1.2 cm-thick with general 55 HU attenuation with small pockets of fluid (22 HU). In the region of the nasal concha, a large area of proliferation was a cause of the external displacement of both eyeballs. The frontal sinuses were affected by osteolysis, and in the region of the right frontal sinus, a large fluid pocket (34 HU) was visible. The region of the left sinus was found to be irregular, with 3.5 × 2.5 × 3.8 cm of new bone formation and a small area of fluid. The brain was unaffected. In the thorax, slight enlargement of the sternal lymph nodes was observed. Within the abdomen, there was a nodule measuring 0.7 cm within the right medial liver lobe. Other abdominal organs were in normal limits.

During the ultrasound examination with using 1–9 MHz microconvex probe (Esaote Twice with Esaote CA123 probe, Eizo Nanao Corp., Firenze, Italy), the soft tissue changes looked similar to those observed by the CT examination. The soft tissues were hypoechogenic and poorly vascularized, with multifocal foci of mineralization. The brain and ocular structures were without visible changes.

**Fig. 2:**
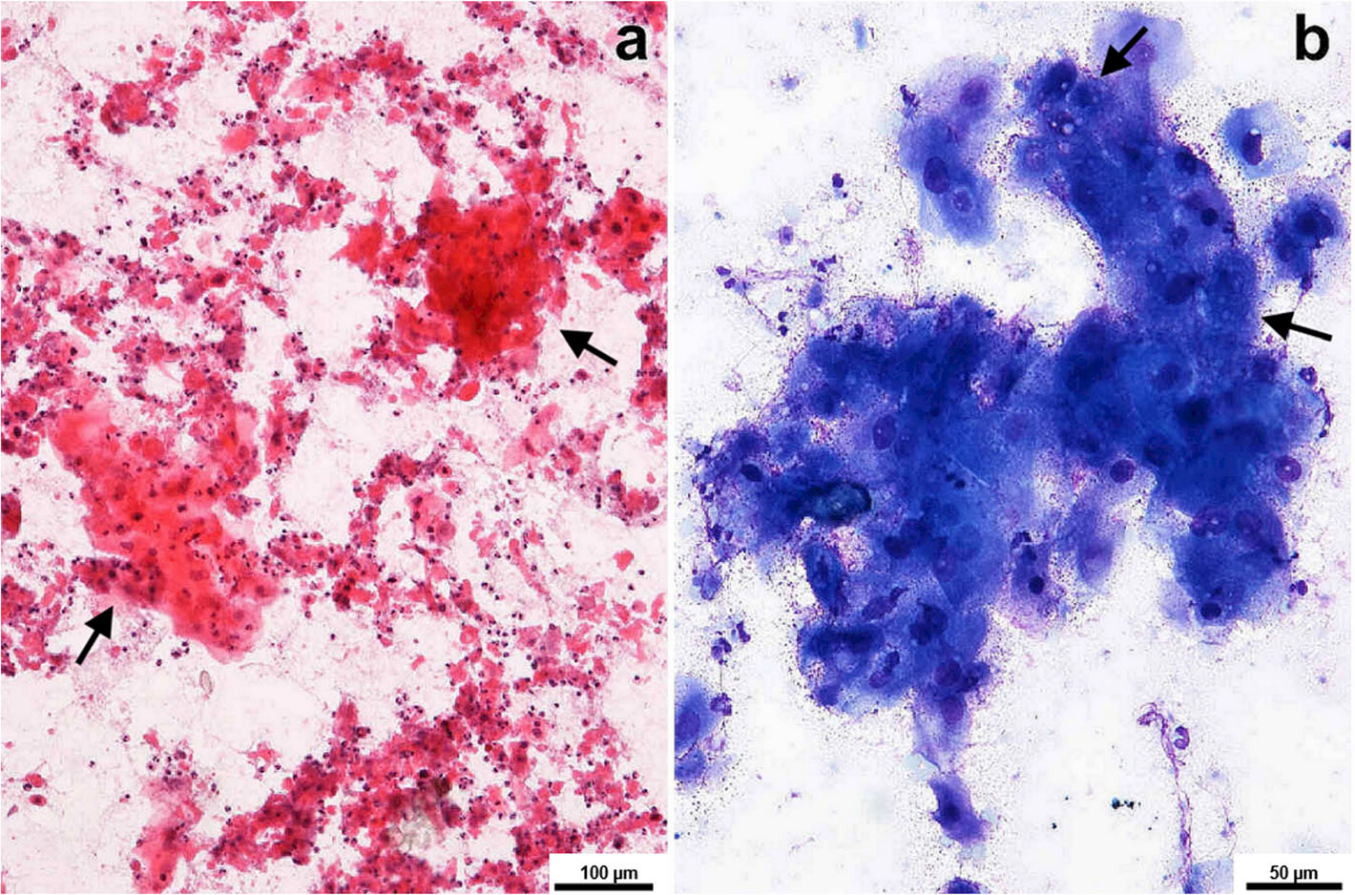
Squamous cell carcinoma – cytology. **a**. Numerous keratinized malignant squamous cells scattered separately or arranged in small clusters (arrows) were dispersed within a background of cellular and karyorrhectic debris (HE, 100 x). **b**. A syncytial aggregate of polygonal squamous cells with abundant basophilic cytoplasm, intracytoplasmic vacuoles (arrows), nuclear size variation and hyperchromatosis (May-Grunwald Giemsa staining, 200 x)

Fine needle aspiration cytology (FNAC) of the lesion in the osteolytic foci of the parietal bone was performed under the same anesthesia event. FNAC smears were sent for cytopathological evaluation at the Department of Pathology. The assessment was made by a trained anatomical pathologist (WŁ). Direct smears were air-dried for May-Grunwald Giemsa staining. Haematoxylin and eosin staining was performed on smears wet-fixed in 95% alcohol. The aspirate smears were densely cellular and contained scattered small clusters of large oval or polygonal, variably keratinized cells with marked anaplasia, hyperchromatic nuclei, and abundant dense cytoplasm frequently containing cytoplasmic vacuoles and showing sharp or straight angles in the periphery, which gave the cells a characteristic flaked look. Many cells contained refractile granules of keratohyalin in the cytoplasm. Karyorrhectic and cellular debris was mixed with inflammatory cells, mainly neutrophils, which were present in the background (Fig. 2). Cytologic diagnosis of SCC was rendered [20].

After the examination, the dog was sent back to the referring physician. The owners did not agree to take any further steps.

Three months after the first visit, the dog re-presented to the clinic. The owners have not reported for any follow-up visit in the meantime. The dog appeared progressively listless, his appetite was decreased, and he had become acutely blind. Consent was obtained for a second set of tomography and blood sampling. Due to the worsening condition of the dog, owners elected humane euthanasia after the CT.

A complete blood count revealed a high WBC and lymphocyte count (34.6 × 10^3^/μl and 10.6 × 10^3^/μl, respectively; reference values 6–16.5 × 10^3^/μl and 1–4.8 × 10^3^/μl respectively) with mild anemia (RBC 4.36 × 10^3^/l, Hb 9.9 d/l, HCT 28%; reference values 5.5–8.5 × 10^3^/l, 12–18 d/l, 37–55%). Total protein and albumin were low (5.49 g/dl and 2.04 g/dl, respectively; reference values 5.5–7.5 g/dl and 3.3–5.6 g/dl), and creatinine and urea were elevated (3.05 mg/dl and 195 mg/dl, respectively; reference values 0.1–1.7 mg/dl and 20–50 mg/dl). The phosphorus and calcium level was mildly elevated as well (8.81 mg/dl and 11.67 mg/dl respectively; reference values 2.5–6.3 mg/dl and 8.4–11.5 mg/dl).

**Fig. 3:**
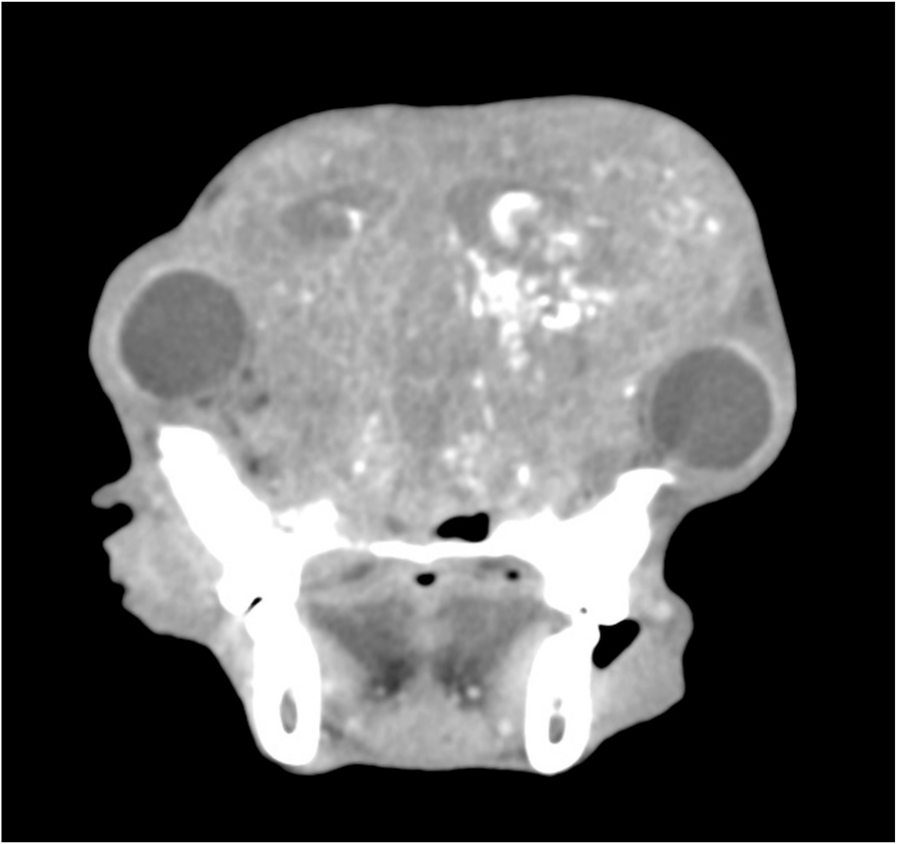
Computed tomographic examination, soft tissue algorithm. There is visible bone extensive osteolysis. Frontal sinuses are fluid- and gas-filled. The retrobulbar spaces were unrecognizable and replaced by new soft tissue formation

**Fig. 4:**
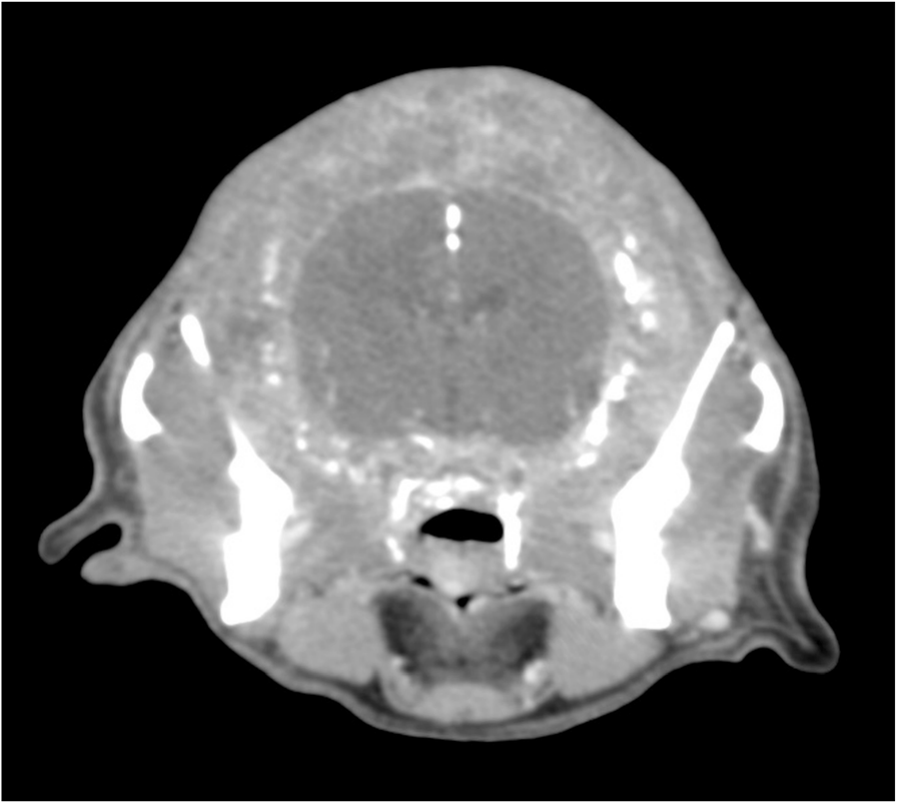
Tomographic examination, soft tissue algorithm. Extensive osteolysis with replacing all bone defects by new soft tissue formation with many small areas of mineralization

CT examination was performed with the previously described protocols. It revealed that very advanced osteolysis affected a significant aspect of the cranium, including the incisive and nasal bone, nasal concha, the palatine bone, the temporal bone, the parietal bone and the occipital bone. There was involvement of the mandible, including the temporal-mandibular joints. The tympanic cavities were fluid-filled. All bone defects had been replaced by new soft tissue formation with multifocal small 1–2 mm areas of mineralization (Fig. 3). The new small tissue formations had a thickness of 3.5 cm. In the brain, small hypodense areas consistent with brain oedema were visible. The frontal sinuses were fluid- and gas-filled, and the previously visible area of new bone formation was affected by osteolytic changes as well. The retrobulbar spaces were unrecognizable and replaced by new soft tissue formation (Fig. 4). There was no visible metastasis within the lungs and thoracic lymph nodes, which were normal in size. In the liver, there was a single visible nodule, as described previously.

**Fig. 5:**
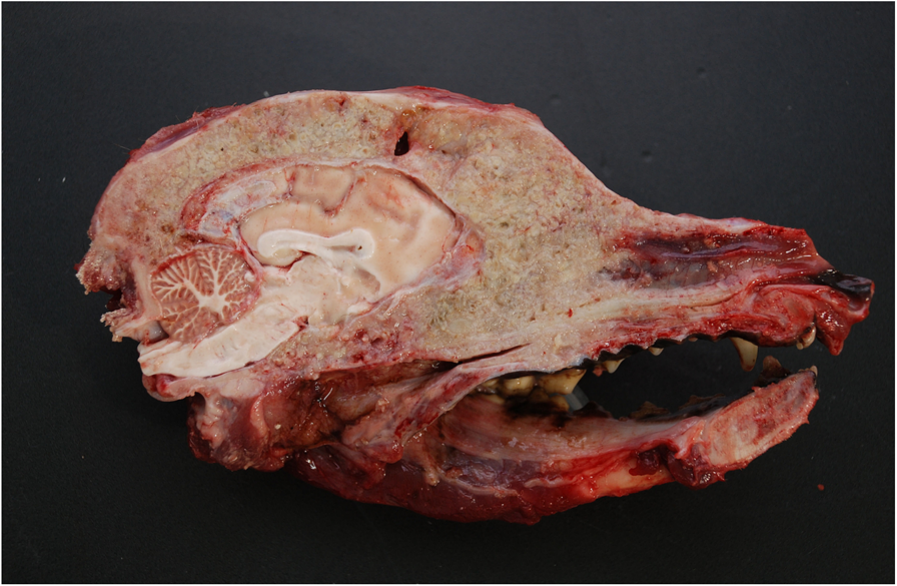
Head. Postmortem view of the medial aspect of the sagittal plane showing/demonstrating extensive thickening and destruction of the skull bones in the parieto-frontal and naso-palatine regions

On humane grounds, the dog was euthanized, and necropsy examination was performed. At necropsy, the gross pathologic findings were limited to the skull. The frontal and occipital regions of the head were significantly enlarged and swollen, while the epidermis covering the skin of the skull was normal. Striking symmetric exophthalmos were observed. After skin and muscle removal, the wrinkled surface of the frontal and parietal bones was visualized. The bones were asymmetrically enlarged (with more prominent deformation on the left side) and easily succumbed to pressure. When cutting the skull along the sagittal plane, it was observed that normal bone was replaced by markedly thickened, softened and deformed bone which indicated an advanced process of bone destruction and remodeling.

(Fig. 5). The interparietal, parietal, and frontal bones, posterior edges of the nasal bone, zygomatic bones and palatine bone were involved. As a result of bone thickening and deformation, the caudal part of the nasal cavity was completely obstructed, and the dimensions of the cranial cavity were reduced, which resulted in increased pressure on the brain structures. In the right lateral hepatic lobe, a single white well-demarcated hard nodule previously visualized by CT was confirmed. No lesions indicative of tumor metastasis was observed within other internal organs. Samples for histopathological examination were obtained from the thickened bones, brain, ocular structures, regional lymph nodes, and internal organs for routine microscopic evaluation. Formalin-fixed, paraffin-embedded tissues were processed for routine microscopy, and the histological sections were stained with haematoxylin and eosin (HE). Bone tissues were decalcified after formalin fixation.

Immunohistochemical staining (IHC) using primary mouse monoclonal antibodies against pan-cytokeratin (PCK) clone AE1/AE3 (Dako, Denmark), diluted 1:100, and Ki-67 antigen clone MIB-1 (Dako, Denmark) diluted 1:100 was carried out to confirm epithelial origin of the tumor and to estimate its proliferative activity and indirectly assess a histologic malignancy. IHC was performed with the use of streptavidinbiotin-peroxidase method (REAL Detection System, Peroxidase/DABţ, Dako, Denmark) according to the manufacturer’s protocol. The immunohistochemical products were visualized by reacting the tissue sections with diaminobenzidine solution (Sigma-Aldrich, Saint-Louis, USA). The sections were counterstained with Mayer’s haematoxylin.

**Fig. 6:**
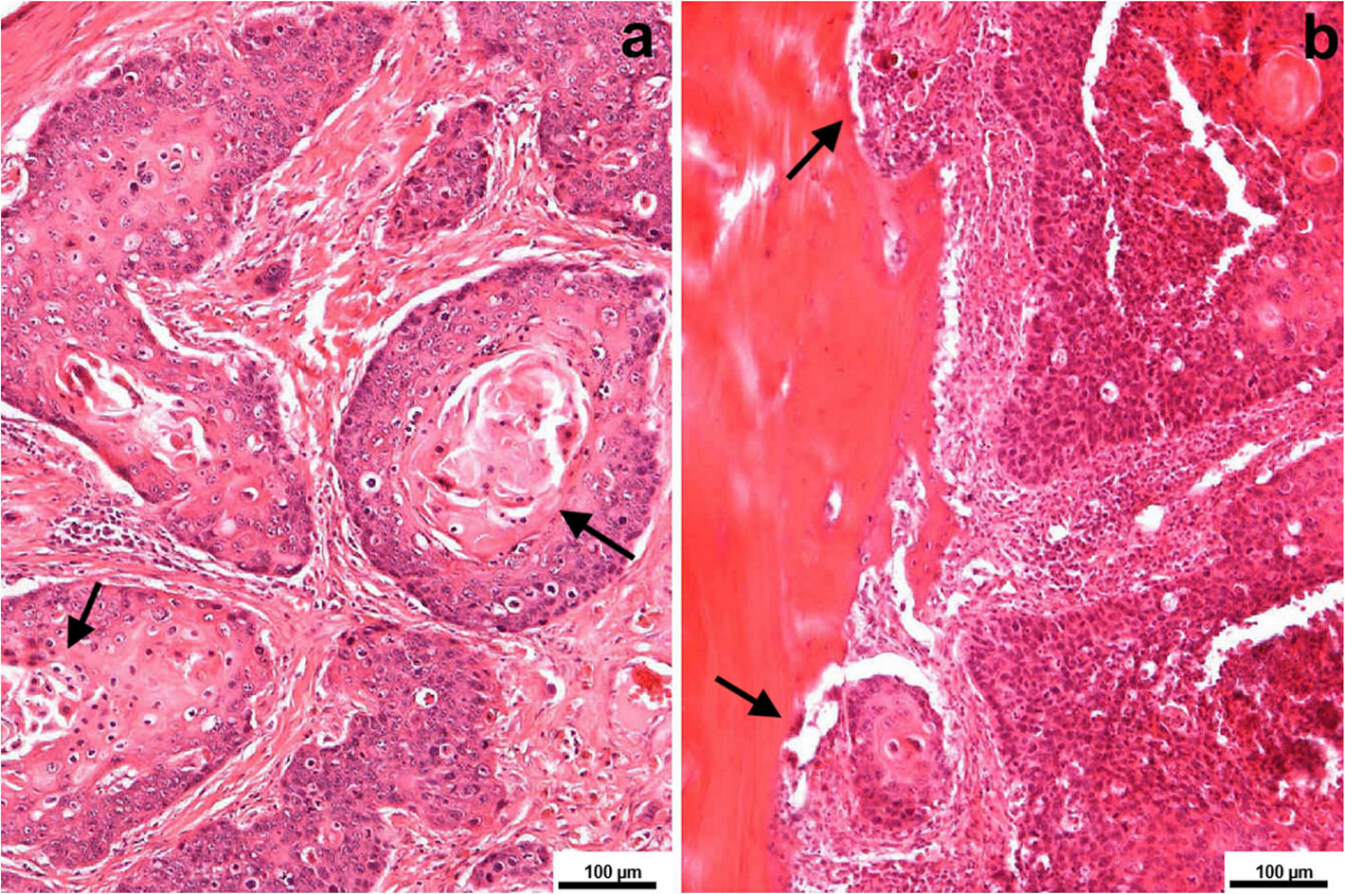
Keratinizing squamous cell carcinoma of the skull. **a** Nests and trabeculae composed of squamous neoplastic cells showing marked pleomorphism. Concentric lamellae of keratin (keratin pearls - arrows) are visible in the center of the larger nest. (HE, 100x). **b** Infiltrative squamous nests invading the bone tissue (arrows) (HE, 100x)

Histopathological examination revealed an unencapsulated infiltrative neoplasms that had expanded, replaced, and infiltrated the entire thickness of the pre-existing skull bones. The covering of the skull epidermis and dermis were not involved. Neoplastic cells of polygonal shape had distinct cell borders with prominent intercellular bridges, abundant eosinophilic cytoplasm and irregular round or oval nuclei with coarsely stippled chromatin and 1–3 magenta nucleoli and were arranged in anastomosing cords, trabeculae and nests supported by a moderate collagenous to desmoplastic stroma. They also exhibited marked anisocytosis and anisokaryosis. The mitotic rate averaged 3 per 40x high power field (HPF) with occasional bizarre mitotic figures. Neoplastic cells frequently exhibited dyskeratosis with brightly eosinophilic cytoplasm and pyknosis. There were multifocal variably sized keratin pearls composed of brightly eosinophilic central accumulation with compact lamellations of keratin (Fig. 6a). Diffusely neoplastic cells invaded and replaced pre-existing bone, in which bony trabeculae were lined by an increased number of osteoclasts in Howship’s lacune, causing diffuse osteolysis. Multifocal reactive bone formation was visible (Fig. 6b). Within the stroma of the neoplasm, there were low numbers of scattered lymphocytes, plasma cells and neutrophils. Neoplastic cells were positive for anti-pan-cytokeratin, and the mitotic index, which was evaluated according to Ki67 (MIB-1) expression, revealed a proliferative rate of up to 28% within the tumor. The described microscopic features were consistent with the diagnosis of well-differentiated keratinizing squamous cell carcinoma with extensive bone invasion, osteolysis and reactive bone formation [20]. All regional lymph nodes were tumor-free. No infiltration of the meninges or brain structures by tumor cells was evident, whereas clefting of perivascular spaces from the surrounding parenchyma suggested brain oedema. The nodule in the liver was diagnosed as a calcified/calcifying parasitic granuloma.

## Discussion and conclusion

SCCs are described in the veterinary literature as a common neoplasia of the cranium, especially in cats, along with mast cell tumors and soft tissue sarcomas [4, 5]. Cutaneous SCC tends to proliferate in the dermis and subcutaneous tissue and show local aggressiveness and infrequent distant metastases; however, in the advanced stages, mild to moderate bone involvement is possible [1, 2, 5, 19]. In people, SCC of the skull has been described as an infiltrating lesion causing adjacent bone destruction but without a tendency to be extensive, especially in cases of papillary SCC and in the upper respiratory tract, for which it constitutes 70–90% of all head malignant lesions [19, 21]. The results of recent studies suggest that malignant transformation of viral papillomas may be caused by different types of canine papilloma viruses, regardless of the location of the lesions in the skin or mucous membranes [22, 23]. Based on epidemiologic data up to 25% of head and neck SCCs in humans are induced by human papillomavirus infection [24]. In our case, the microscopic examination did not reveal any features typical of a viral infection, i.e. intranuclear inclusion bodies, koilocyte formation or large clumped keratohyalin granule accumulation, however this does not exclude viral etiology. Identification of viral particles in pathogenesis of SCC can be attempted by screening of the tissue with polymerase chain reaction or immunohistochemistry [16, 17]. However, these techniques are not readily available in practice and were not performed in our case.

In animals, the possibility of the local aggressiveness of SCCs has been mentioned only in a few reports [2, 25]. Tomographic findings include the presence of neoplastic tissue with areas of mineralization and variable osteolysis of adjacent bone [5, 8, 19]. New bone production is rare [5].

The manifestations of the case presented in this paper differ significantly from the typical manifestations of previously described skull SCC in animals, as the extensive and generalized degree of bone destruction is not typical for cutaneous epithelial neoplasia [7, 19]. Based on our knowledge, no similar cases of cranial squamous cell carcinoma in dogs with such aggressive and extended bone infiltration and destruction have been described. In the veterinary literature, we have found only one case report with a very similar appearance in a cat with a diagnosis of adenosquamous carcinoma (AC) [12]. This case was the first case of a neoplasia in a cat, which involved most anatomical cranial structures with features very similar to those of our case. The lesions in this cat probably originated from the nasal cavity and paranasal sinuses. This location was interesting for the authors to discuss because most inflammatory and neoplastic processes of the nasal cavity and paranasal sinuses in animals are only regional and do not involve other anatomic cranial structures, which was different from the Chow et al. case [12, 26, 27]. In our dog, the first examination showed that the nasal cavity was not the primary location of the described lesions, as Chow et al. suspected in their case. The epicenter of lysis and new tissue formation were observed to be primarily in the frontal and parietal bones in the area of the frontal sinuses. This is one of the rarely reported locations of squamous cell carcinomas in animals. Next, the process extended cranially and caudally to include the nasal bones. Despite the extent of the process, as in the case reported by Chow et al., distant metastases were not found, and the process was only regional. The histological appearance was different; glandular tissue was not observed in our specimens, and only keratinization foci were observed.

Interestingly, in human medicine, variants of tumors arising from squamous epithelium or resembling SCC have been described in the skull. They include adenosquamous carcinoma and benign and malignant proliferating trichilemmal tumors in addition to SCC [7, 13, 28, 29]. Their differentiation is based on careful microscopic analysis and is sometimes also supported by immunohistochemistry [28].

Adenosquamous carcinoma has been described in the literature as a rare, highly aggressive neoplasia with histological features of SCC and AC [12–14]. It has been described in many body areas, including the thorax and abdomen, and less frequently in the larynx, oral cavity, nasal cavity, pharynx, with a tendency towards perineural invasion [12–14, 18, 19]. In animals, it has been reported in the esophagus and nasal cavity, as in the Chow et al. case [12, 13]. In human medicine, head and neck localization of AC has been broadly discussed, and this type of tumor has been considered to be a mucoepidermoid carcinoma in some reports [13, 14, 18].

Another type of tumor, proliferating trichilemmal tumor (PTT), is a rare, often benign neoplasm arising from the isthmus region of the outer root sheath of the hair follicle. In humans, it occurs most commonly on the face and scalp region [3, 30, 31]. The histological hallmark of PTT is the presence of abrupt trichilemmal keratinization, which is the sudden transition of a nucleated epithelial cell to an anucleated, amorphous, compact keratinized cell and is an important histological finding helping to differentiate it from SCC [3, 30, 32]. CD34 and calretinin are two important immunohistochemical markers of outer root sheath differentiation that show positivity in PTT. Recently, PTT was classified into three types: benign, locally aggressive, and malignant [33]. The risk of metastasis increases when the tumor involves an ear, has a thickness above 2 mm or has a diameter above 6 mm [7]. Bone invasion is uncommon, but there have been a few reports of cranial invasion without metastasis, although the risk according to the abovementioned guidelines is high [7]. In other cases, perineurial infiltration has been observed, including in the cranium dura and brain parenchyma [7, 9, 34, 35].

PTT has been reported as a complication after head trauma and inflammation, with slow growth [3, 30]. It is similar to that observed in our patient, although head trauma was only the owner’s suspicion, and there was no proof that it had occurred [3]. The first clinical signs that were observed were a bruise on the forehead and dullness. Similar signs (crusting, erythema, superficial erosion and ulcers) are also the first clinical signs observed in squamous cell carcinoma [1]. According to the owner’s history, the growth of the lesion was very rapid. The dog was maintained outdoors and allowed to run free on the property, so it is possible that owners did not observe the actual first symptoms.

The classification of SCC and possible variants of tumors arising from the squamous epithelium described in literature show that the manifestations of the disease can vary. As in the research of Chow et al., we do not have any confirmation of immunosuppression or viral disease in our case, which could be a reason for the viral etiology of the lesion and could lead to aggressive infiltration and destruction of the surrounding tissues. According to the medical literature, this possibility should be the subject of further research in cases of advanced malignant bone osteolysis.

In conclusion, on the basis of our described case, which is the only case that we found in the veterinary literature with similar clinical manifestations, with particular emphasis on extensive osteolysis unique for this type of tumor and the diagnostic imaging changes caused by other cranial neoplasms in animals, it is justified to suspect a process of neoplastic squamous origin in all cases of aggressive and extensive bone lysis. This issue should be a topic of further investigation.

## Data Availability

The datasets used and/or analyzed during the current study are available from the corresponding author on reasonable request.

## Abbreviations

SCC: Squamous cell carcinoma
HE: Haematoxylin and eosin
FNAC: Fine needle aspiration cytology
AC: Adenosquamous carcinoma
PTT: Proliferating trichilemmal tumor
PCK: Pancytokeratin
IHC: Immunohistochemistry
